# Cell-free DNA comparative analysis of the genomic landscape of first-line hormone receptor-positive metastatic breast cancer from the US and China

**DOI:** 10.1007/s10549-021-06370-w

**Published:** 2021-09-01

**Authors:** Xiaoran Liu, Andrew A. Davis, Feng Xie, Xinyu Gui, Yifei Chen, Qiang Zhang, Lorenzo Gerratana, Youbin Zhang, Ami N. Shah, Amir Behdad, Firas Wehbe, Yong Huang, Jianjun Yu, Pan Du, Shidong Jia, Huiping Li, Massimo Cristofanilli

**Affiliations:** 1 Key Laboratory of Carcinogenesis and Translational Research (Ministry of Education/Beijing), Department of Breast Oncology, Peking University Cancer Hospital & Institute, Fu-Cheng road No. 52, Hai-Dian District, Beijing, 100142 China; 2 Robert H. Lurie Comprehensive Cancer Center, Feinberg School of Medicine, Northwestern University, Chicago, IL USA; 3 Department of Medicine, Division of Oncology, Washington University School of Medicine in St. Louis, St. Louis, MO USA; 4 Huidu (Shanghai) Medical Sciences, Ltd., Shanghai, China; 5 Predicine, Inc., Hayward, CA USA

**Keywords:** HR-positive advanced breast cancer, Circulating tumor DNA, Next-generation sequencing

## Abstract

**Purpose:**

Meaningful comparison of mutational landscapes across ethnic groups requires the use of standardized platform technology. We have used a harmonized NGS-based liquid biopsy assay to explore the differential genomic landscape of patients with initially hormone receptor-positive (HR+), HER2-negative MBC of first line metastasis or primary Stage IV at diagnosis from the United States (US) and China (CN).

**Methods:**

Plasma circulating tumor DNA (ctDNA) from 27 US patients and 65 CN patients was sequenced using the harmonized CLIA-certified, 152-gene PredicineCare™ liquid biopsy assay. Kaplan–Meier survival analysis was performed to analyze the correlation between genomic alterations and progression-free survival (PFS), and *p*-values were calculated using the log-rank test.

**Results:**

All patients in the CN cohort received chemotherapy and/or hormonal therapy, while 85.2% (23/27) patients in the US cohort received hormonal therapy plus CDK4/6 inhibitors. Mutations were detected in 23 of 27 (85%) US patients and 54 of 65 (83%) CN patients. The prevalence of *AKT1* (*P* = 0.008) and *CDH1* (*P* = 0.021) alterations were both higher in the US vs. CN cohort. In addition, *FGFR1* amplification were more frequent in the CN vs. US cohort (*P* = 0.048). *PTEN* deletions (*P* = 0.03) and *ESR1* alterations (*P* = 0.02) were associated with shorter PFS in the CN cohort, neither of these associations were observed in the US cohort. Interestingly, a reduced association between *PTEN* deletion and PFS was observed in patients receiving CDK4/6 inhibitor treatment.

**Conclusion:**

The differential prevalence of ctDNA-based alterations such as *FGFR1*, *AKT1*, and *CDH1* was observed in initially HR+/HER2− MBC patients in the US vs. CN. In addition, the association of *PTEN* deletions with shorter PFS was found in the CN but not the US cohort. The differential genomic landscapes across the two ethnic groups may reflect biologic differences and clinical implications.

**Supplementary Information:**

The online version contains supplementary material available at 10.1007/s10549-021-06370-w.

## Introduction

Metastatic breast cancer (MBC) is a heterogeneous disease with increased genomic complexity compared to primary breast cancer and is associated with known somatic mutations, which vary across different subtypes [1]. Hormone receptor-positive (HR+), HER2-negative (HER2−) breast cancer (BC) accounts for over 70% percent of all breast cancer cases [2, 3]. Currently, endocrine therapy, CDK4/6 inhibitors, and PI3K/AKT/mTOR pathway inhibitors are being used in the clinic with several novel endocrine, targeted, and chemotherapy-based treatments in clinical development [4, 5].

Liquid biopsies, including cell-free DNA (cfDNA), circulating tumor DNA (ctDNA), cell-free RNA, circulating tumor cells, exosomes, and protein, have emerged as clinically relevant analytes for determining prognosis, genomic characterization, and therapeutic response in MBC. For MBC patients with metastasis to lung, liver, or bone acquiring biopsies, particularly at serial timepoints, can be difficult and invasive [6]. Furthermore, tissue biopsies only capture a spatially and temporally limited snapshot of disease biology [7]. In contrast, cfDNA assays provide non-invasive tools that facilitate serial disease monitoring and capture tumor heterogeneity. In multiple clinical trials for patients with MBC, ctDNA mutational profiling has been incorporated into the drug development process to identify potential predictive biomarkers [8]. For example, in one trail *ESR1* mutations were present in 37% of baseline samples and were enriched in patients with luminal A and *PIK3CA*-mutated tumors [9]. Another study using archived baseline plasma from the SoFEA and PALOMA-3 trails reported that *ESR1* mutations were present in 39.1% of patients, respectively. Analysis of cfDNA from the BOLERO-2 trial using droplet digital PCR (ddPCR) showed that 28.8% of patients had either D538G or Y537S mutations in *ESR1* [10] and 43.3% of the patients harbored H1047R, E545K, or E542K mutations in *PIK3CA* [11]. Another study, using whole exome sequencing of plasma DNA from the PALOMA-3 trial, revealed dynamic changes in *PIK3CA* and *ESR1* mutations after treatment, demonstrating the utility of ctDNA assays for serial monitoring in MBC [12, 13].

Despite diagnostic and therapeutic advances for patients with MBC, breast cancer patients experience dramatic differences in terms of disease onset, management, and clinical outcomes between CN and western countries. For instance, the median age of diagnosis in Chinese breast cancer is almost 10 years younger than in the United States (US) and in European Union (EU) [14]. Genomic profiling of breast cancer patients in the US vs. CN has generated conflicting results. While some studies have reported significant differences in certain gene pathways and molecular subtypes [15–19], others have described similar genetic landscape [20, 21].

Few studies have explored the differences in genomic features of tumors, particularly based on ctDNA assessment, across these populations. In this study, we used a harmonized ctDNA liquid biopsy test to systematically compare the genomic landscape of patients with first relapse of distant metastasis after surgery or those with primary stage IV metastatic diagnosis in the US and CN. All the patients have initially HR+/HER2− tumors.

## Materials and methods

### Patients

Two cohorts with initially HR+/HER2− BC at the time of first-line metastatic breast cancer (first-line MBC) or primary stage IV breast cancer (primary stage IV BC) pathological diagnosis from the US (February 2017–October 2019) and CN (March 2018–March 2019) participated in this study. The enrollment criteria were as follows: (1) The patients had relapse after surgery or primary stage IV BC. (2) A diagnosis of HR+ and HER2− of primary tumor. HR+ was defined as ≥ 1% positive tumor nuclear estrogen receptor (ER) and/or progesterone receptor (PR) [22]. HER2 status was determined via immunohistochemistry (range 0–3+). For HER2 results, 0 and 1+ were classified as negative, 2+ as equivocal, and 3+ as positive. Fluorescence in situ hybridization tests were used to confirm HER2 status when immunohistochemistry results were equivocal. (3) Patients signed informed consent for additional blood to be collected for gene testing. (4) Age between 18 and 85 years. (5) At least one measurable lesion according to the Response Evaluation Criteria in Solid Tumors (RECIST) version 1.1. (6) Patients had a performance status score of Eastern Cooperative Oncology Group (ECOG) ≤ 1. (7) Patients had a life expectancy of at least 12 weeks and adequate hematologic, hepatic, and renal function. Plasma samples of 10 mL were prospectively collected from each patient. The samples were collected at the time of diagnosis of first-line MBC or primary stage IV BC and before treatment. Institutional Review Boards at each site approved the study in the US (ethic No. NU16B06) and CN (ethic No. 2016KT75).

### Plasma cfDNA NGS testing

cfDNA testing from plasma was performed using the harmonized 152-gene PredicineCARE assay in two Colleges of American Pathologist (CAP)-accredited laboratories separately, one was in the US (Predicine Inc.) and another in China (Huidu Shanghai Medical Sciences Ltd.). The final comparative data analysis was conducted in China. The detailed information of 152-gene PredicineCARE can be found in supplementary Table 2.

### Plasma cfDNA extraction

cfDNA was extracted from plasma samples using QIAamp circulating nucleic acid kit. Quantity and quality of the purified cfDNA were checked using Qubit fluorimeter and Bioanalyzer 2100.

### Library preparation, capture, and sequencing

5 to 20 ng of extracted cfDNA was prepared for library construction including end-repair, dA-tailing, adapter ligation, and PCR amplification. The amplified DNA libraries with sufficient yields proceeded to hybrid capture. In brief, the library was hybridized overnight with the panel probes. Unbound fragments were then washed away. The purified libraries were QCed with Bioanalyzer 2100 and then paired-end 2 × 150 bp sequenced using the Illumina sequencing platform.

### Variant calling

Variants were called using a Predicine in-house analysis pipeline, starting from the raw sequencing data to the final mutation calls, which has been described in previous publications [23, 24]. Briefly, the pipeline first performed adapter trimming, barcode checking, and correction. Cleaned paired FASTQ files were outputted by the in-house pipeline and further aligned to the human reference genome build hg19 using BWA (version 0.7.15) alignment tool. Consensus bam files were then derived by merging paired-end reads originated from the same molecules (based on mapping location and unique molecular identifiers) as single-strand fragments. Single-strand fragments from the same double-strand DNA molecules were further merged as double stranded. Both sequencing and PCR errors were deeply suppressed during this process. Candidate variants, consisting of point mutations, small insertions, and deletions, were identified across the targeted regions covered in the panel. Copy number variations were estimated at the gene level. The pipeline calculated the on-target unique fragment coverage, which was first corrected for GC bias and was then adjusted to the probe level bias (estimated from a pooled reference).

### Statistical analysis

Fisher’s exact test was performed to compare the mutational prevalence between CN and US cohorts. The basic clinical characteristic comparison of the two cohorts were carried out using a *t* test. Kaplan–Meier survival analysis was performed to analyze the correlation between genomic alterations and progression-free survival (PFS), and *P*-values were calculated using the log-rank test by comparing the patients with and without a particular genomic alteration. R (version 3.5) was used for statistical analysis. Survival and Survminer R packages were used for survival analysis.

## Results

### Baseline patient characteristics

In total, there were 27 US patients at Northwestern University and 65 Chinese patients at Peking University Cancer Hospital enrolled in this study based on the inclusion criteria (see Materials and Methods). All were female. All patients were confirmed for a diagnosis of initially HR+/HER2− tumors. HR and HER2 status of metastatic tumors were confirmed for twenty-one patients from the CN cohort, with 18 patients maintaining HR+ and HER2− status, while 3 patients became HR- and HER2−. In the US cohort, the HR and HER2 status of metastatic tumor was confirmed for 23 patients, with all patients maintaining HR+ and HER2− status. Median age of diagnosis was 51 years (range: 30–79) in the US cohort and 49 years (range: 27–82) in the CN cohort. In the US cohort, 4 out of 27 (14.8%) patients were primary stage IV BC and the rest (23/27, 85.2%) were all first-line MBC. In CN cohort, 15 out of 65 (23.1%) patients were primary stage IV BC and the rest (50/65, 76.9%) were all first-line MBC. The sites of metastasis in decreasing order of frequency were liver, lung, and bone in the US cohort and lung, liver, and bone in the CN cohort. There were no significant differences found between US and Chinese samples with respect to age of diagnosis and primary tumor stages (mostly stage II or above in US and Chinese patients, 91.3% and 75.0%, respectively). Patients’ clinical and pathological characteristics are summarized in Table 1.

**Table 1:** Comparison of baseline clinical characteristics between the US and CN (*n* = 90)

Clinical characteristics	US (*n* = 27)	CN (*n* = 65)	*P*
Age at diagnosis			0.400
≤ 45 years	10 (37.0%)	21 (31.7%)	
> 45 years	17 (63.0%)	44 (68.3%)	
HR intensity of primary tumor			0.005
≤ 25%	9 (33.3%)	7 (9.5%)	
> 25%	18 (66.7%)	58 (90.5%)	
Tumor grade of primary tumor			0.306
I	2 (7.4%)	4 (6.2%)	
II	13 (48.1%)	32 (49.2%)	
III	8 (29.6%)	10 (15.4%)	
Unknown	4(14.8%)	19(29.2%)	
T stage of primary tumor			0.013
T1/2	15 (55.6%)	52 (80.0%)	
T3/4	10 (37.0%)	8 (12.3%)	
Unknown	2 (7.4%)	5 (7.7%)	
N stage of primary tumor			0.776
N0/1	14 (51.6%)	33 (50.8%)	
N2/3	12 (44.4%)	27 (41.5%)	
Unknown	1 (3.7%)	5 (7.7%)	
Previous neoadjuvant chemotherapy			0.022
No	11 (40.8%)	54 (83.1%)	
Yes	9 (33.3%)	11 (16.9%)	
Unknown	7 (25.9%)	0 (0.0%)	
Previous adjuvant chemotherapy			0.987
No	8 (29.6%)	24 (36.9%)	
Yes	15 (55.5%)	39 (60.0%)	
Unknown	4 (14.8%)	2 (3.1%)	
Previous adjuvant endocrine therapy			< 0.001
No	4 (14.8%)	20 (30.8%)	
SERM^a^	8 (29.6%)	33 (50.8%)	
AI^b^	8 (29.6%)	10 (15.4%)	
SERM+AI	5 (18.5%)	1 (1.5%)	
Unknown	2 (7.5%)	1 (1.5%)	
Disease-free survival			0.161
≤ 36.0 months	9 (33.3%)	11 (16.9%)	
> 36.0 months	14 (51.9%)	39 (60.0%)	
Primary stage IV	4 (14.8%)	15 (23.1%)	
Liver metastasis			0.129
No	24 (88.9%)	50 (75.8%)	
Yes	3 (11.1%)	15 (24.2%)	
Lung metastasis			0.021
No	23 (85.2%)	40 (61.3%)	
Yes	4 (14.8%)	25 (38.7%)	
Brain metastasis			0.217
No	25 (92.6%)	64 (98.4%)	
Yes	2 (7.4%)	1 (1.6%)	
Bone metastasis			0.538
No	10 (37.0%)	25 (38.7%)	
Yes	17 (63.0%)	40 (61.3%)	
Lymph metastasis			0.078
No	16 (59.3%)	27 (40.3%)	
Yes	11 (40.7%)	38 (59.7%)	
Chest metastasis			0.156
No	27 (100.0%)	60 (91.9%)	
Yes	0 (0.0%)	5 (8.1%)	
Other metastasis			0.386
No	19 (70.4%)	48 (75.8%)	
Yes	8 (29.6%)	17 (24.2%)	
Liver and/or lung metastasis			0.021
No	20 (74.1%)	31 (48.4%)	
Yes	7 (25.9%)	34 (51.6%)	
First-line therapeutic regimen			0.000
Chemotherapy	0 (0.0%)	22 (33.8%)	
Hormonal therapy	3 (11.1%)	15 (23.1%)	
Chemotherapy followed by hormonal therapy	0 (0.0%)	28 (43.1%)	
Hormonal plus CDK4/6 inhibitor	23 (85.2%)	0 (0.0%)	
Unknown	1 (3.7%)	0 (0.0%)	

^a^SERM: tamoxifen/toremifene

^b^AI: anastrozole/exemestane/letrozole

### Mutational and CNV landscape

**Fig. 1:**
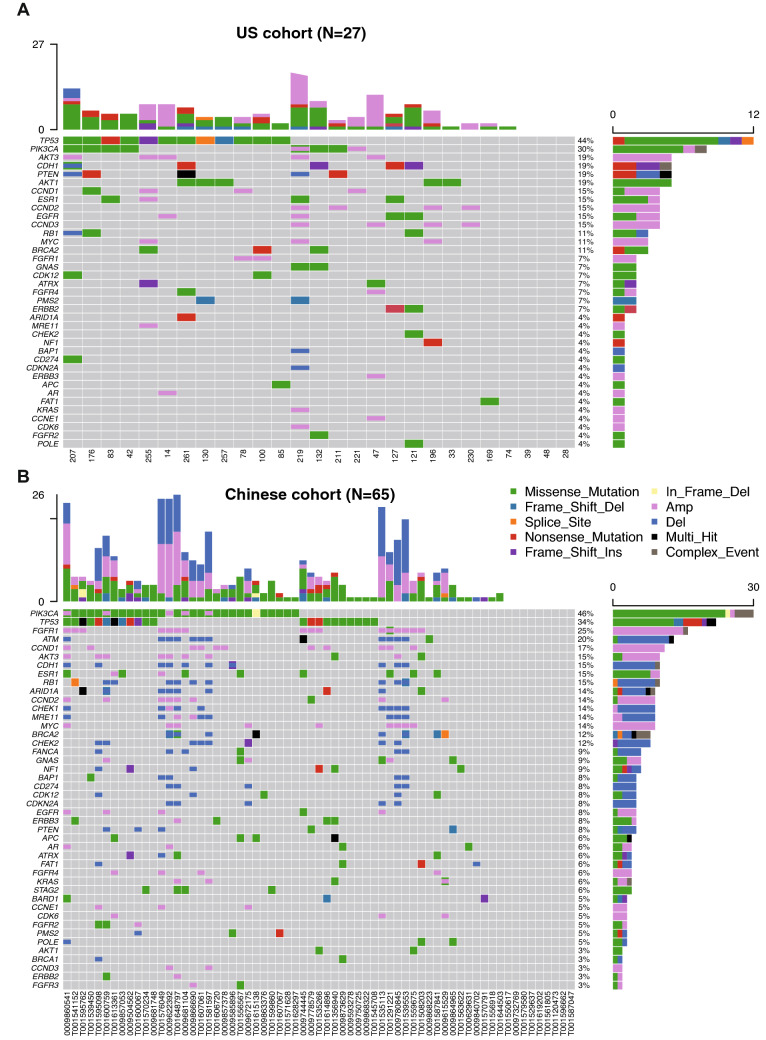

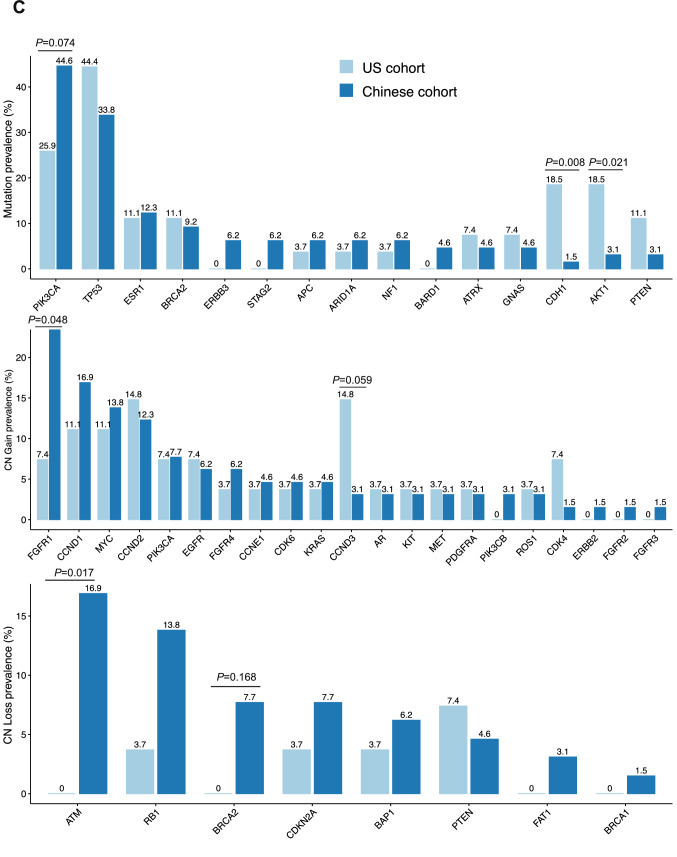
ctDNA mutation landscapes of US and Chinese HR+ MBC patients. **A** Mutation profile of US cohort. **B** Mutation profile of Chinese cohort. **C** Prevalence of gene mutation, copy number (CN) gain, and CN loss in US and Chinese cohorts. *P* value was calculated using one-side Fisher’s exact test

**Fig. 2:**
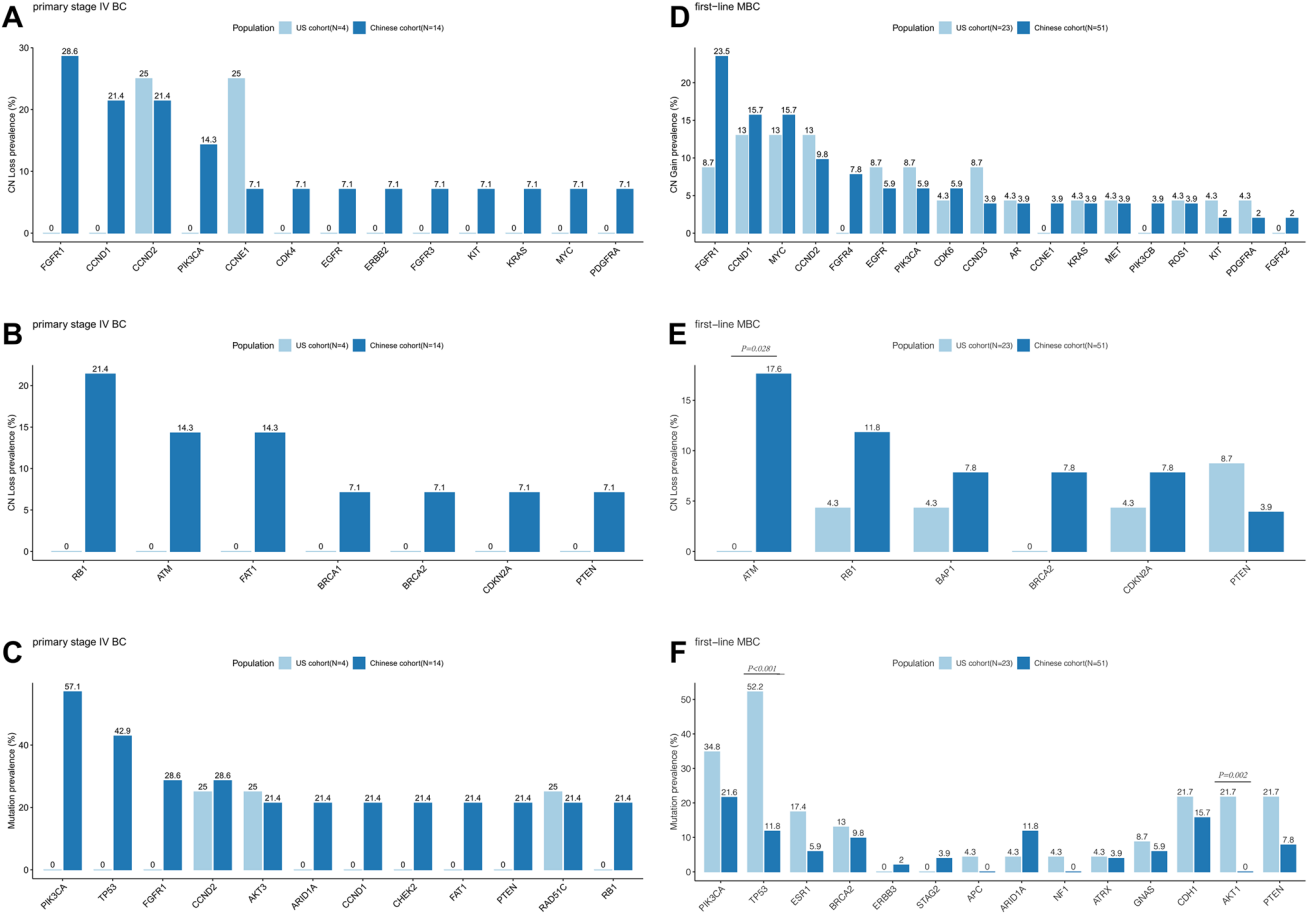
Subgroup analysis of differential mutation prevalence between US and CN patients. **A**, **B**, **C** Comparison of mutation prevalence between US and CN patients in primary stage IV BC subgroup; **D**, **E**, **F** Comparison of mutation prevalence between US and CN patients in first-line MBC subgroup. Mutations which detected more than twice in US or CN cohort were included for analysis. Comparison between groups with statistical significance were marked by corresponding *P* values above

The samples were tested using a harmonized NGS-based 152-gene PredicineCARE™ assay at Predicine Inc. in the US and at Huidu (Shanghai) in CN. SNV/Indel mutations were detected in 23 of 27 (85%) US patients and 54 of 65 (83%) Chinese patients. The number of mutations detected in each patient ranged from 0 to 9 in US patients and 0 to 9 in Chinese patients. CNVs, including both copy number gain and loss, were also detected in 13 of 27 (48%) US patients and 32 of 65 (51%) Chinese patients. The number of CNVs detected in each patient ranged from 0 to 19 in the US patients and 0 to 24 in the Chinese patients. The most frequently detected SNV/Indel mutations included *TP53* (44.6%), *PIK3CA* (25.9%), *ESR1 *(11.1%), and *BRCA2* (11.1%) in US patients vs. *TP53 *(33.8%), *PIK3CA* (44.6%), *ESR1* (12.3%), and *BRCA2* (9.2%) in Chinese patients. Alterations in *CDH1 *(18.5% vs. 1.5%, *P* = 0.008) and *AKT1* (18.5% vs. 3.1%, *P* = 0.021) were more frequent in the US cohort than in the CN cohort. However, the prevalence of *PIK3CA* alteration between US and CN patients was not observed in total cohort (25.9 vs. 44.6%, *P* = 0.074). The most frequently detected CNVs included copy number gains of the following genes in the US vs. CN patients: *CCND1* (11.1 vs 16.9%), *CCND2* (14.8 vs 12.3%), *CCND3* (14.8 vs. 3.1%), and *MYC* (11.1 vs. 13.8%), respectively. There were no statistically significant differences in these genomic alterations. In contrast, *FGFR1* copy number gain was significantly different between the US (7.4%) and CN (24.6%) (*P* = 0.048). *CDK4* copy number gain was not significantly different between the US (7.4%) and CN (1.5%) populations (*P* = 0.205). No significant differences were observed for most copy number loss alterations in the US vs. CN patients except *ATM*: *ATM* loss (0 vs. 16.9%, *P* = 0.017), *RB1* loss (3.7 vs. 13.8%, *P* = 0.145), *BRCA2* loss (0 vs 7.7%, *P* = 0.168), *PTEN* (7.4 vs. 4.6%, *P* = 0.460), and *BRCA1* (0 vs 1.5%, *P* = 0.707) (Fig. 1A–C). In first-line MBC subgroup, *TP53* (52.2 vs. 11.8%, *P* < 0.001) and *AKT* (0 vs. 21.7%, *P* = 0.002) mutations were more frequently found in US as compared to CN cohort. *ATM* loss (0 vs. 17.6%, *P* = 0.028) was more frequently found in CN than in US patients. However, the differential prevalence of *PIK3CA* mutation (*P* = 0.179) and *FGFR1* copy number gain (*P* = 0.115) between US and CN patients were not found in this subgroup. (Fig. 2A–C). In primary stage IV BC subgroup, no significant differential prevalence of alterations was found between US and CN cohort. (Fig. 2E, F).

### Survival analysis

**Fig. 3:**
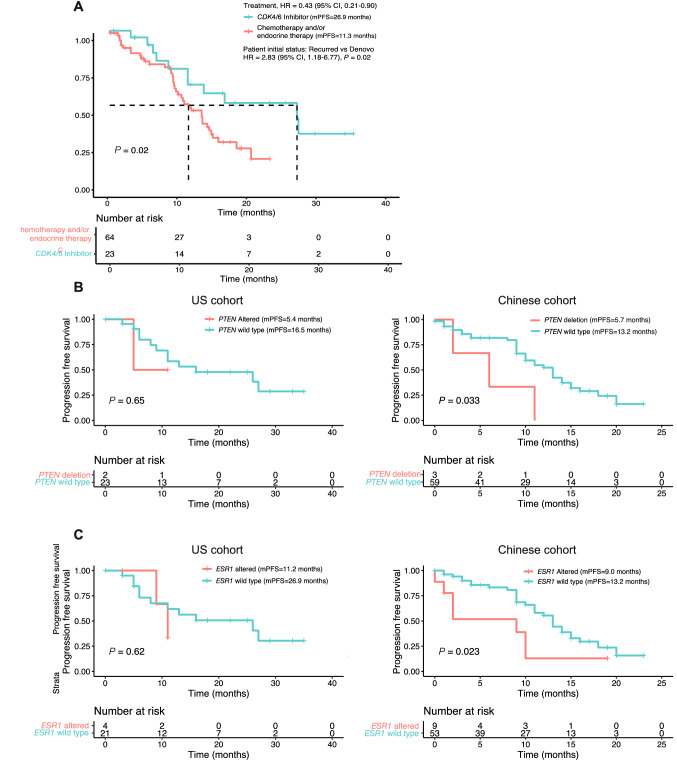
Survival analysis of Chinese and US patients. **A** Prolonged progression-free survival (PFS) in patients treated with CDK4/6 inhibitor. HR, hazard ratio, CI, confidence interval. **B** PFS of patients with or without PTEN detection. **C** PFS of patients with or without ESR1 activating mutation or copy number gain. *P* value was calculated using log-rank test

The first-line treatment regimens for US and CN patients were different. The majority of patients (85.2%) in the US cohort were treated with standard-of-care single hormonal therapy plus CDK4/6 inhibitor treatment, while no patients in the Chinese cohort received upfront CDK4/6 inhibitors. In the total cohort, patients who received hormonal therapy plus CDK4/6 inhibitors were set up as one group and the PFS of this group was compared to the group receiving hormonal therapy only. Multivariate analysis showed that patients receiving hormonal therapy plus CDK4/6 inhibitors had longer median PFS compared to those receiving hormonal therapy alone (26.9 vs. 11.3 months, *P* = 0.025) (Fig. 3A). We also carried out survival analysis for patients with any *AKT*-activating, *PTEN*-inactivating, or DDR deficiency mutations, in the Chinese and US cohorts. (Supplementary Fig. 1, and data not shown). *PTEN* deletion was associated with shorter PFS of HR+ MBC patients in CN (5.7 vs. 13.2 months, *P* = 0.03) (Fig. 3B). This result was not encountered in US patients (5.4 vs. 16.5 months, *P* = 0.65) (Fig. 3B). The same result regarding *PTEN* deletion was also observed in multivariate analysis considering recurrent/initial stage IV status of the patients (Supplementary Table 1). Meanwhile, patients with cfDNA-based *ESR1* alterations had shorter PFS compared to the other patients in the first-line treatment after relapse in the CN patient cohort (9.0 months vs. 13.2 months, *P* = 0.023) (Fig. 3C). A similar trend was not statistically significant in US patients (11.2 vs. 26.9 months, *P* = 0.62) (Fig. 3C). The same result regarding *ESR1* alteration was also observed in multivariate analysis considering recurrent/initial stage IV status of the patients (Supplementary Table 1). cfDNA yield has previously been reported as a prognostic biomarker related to patient disease progression and prognosis [25]. However, there was no significant difference in cfDNA yield between Chinese and US patients in this study. (Supplementary Fig. 2). In addition to mutational aspect, we also found that patients with liver or lung metastasis tend to have shorter PFS (14.3 vs. 10.5 months, *P* = 0.15) (Data not showed). This trend was observed in the CN cohort (14.3 vs. 10.3 months, *P* = 0.18) but not in the US cohort (13.5 vs. 16.5 months, *P* = 0.80). (Data not showed).

## Discussion

**Fig. 4:**
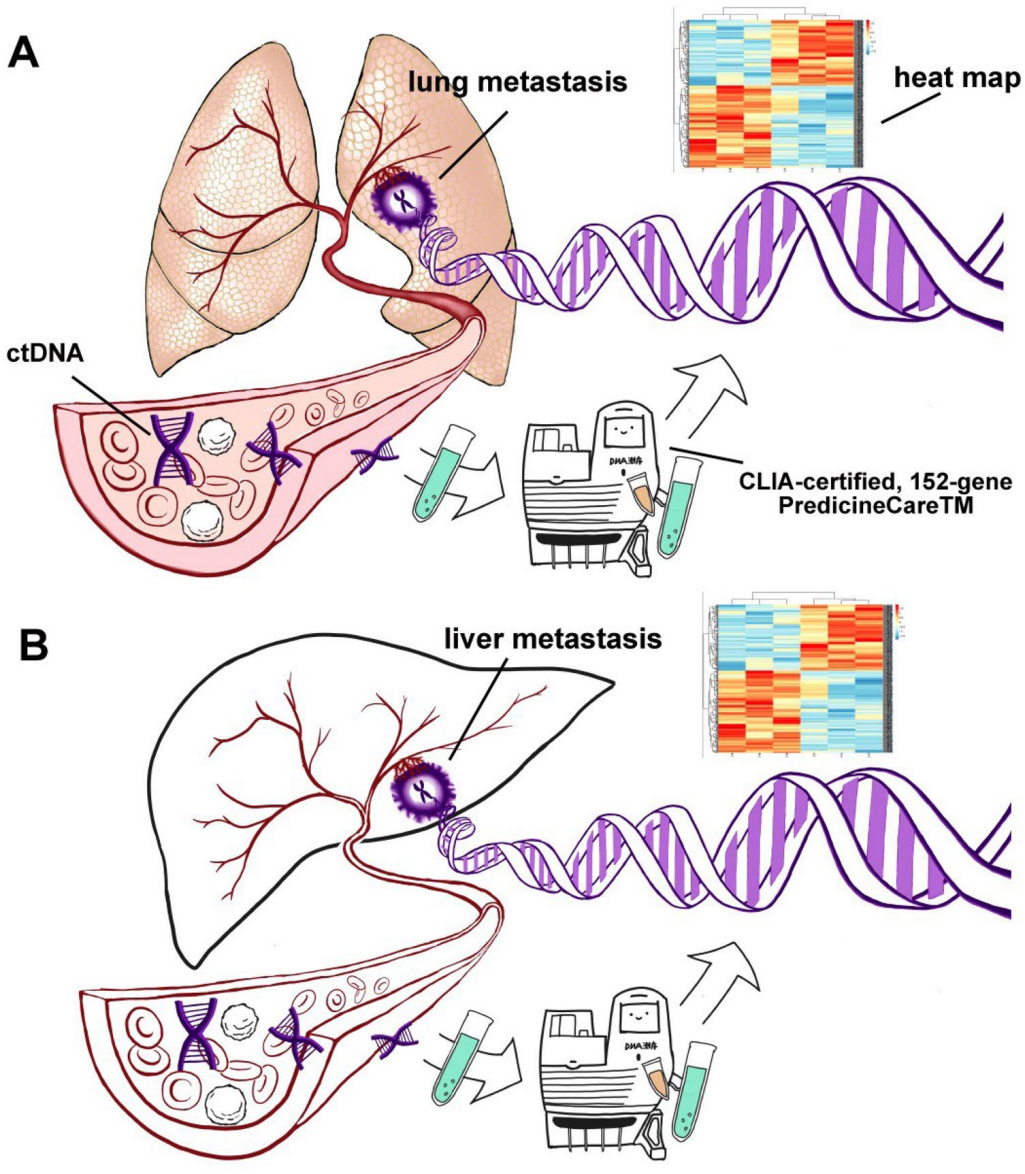
Schematic diagram of ctDNA detection. **A** Detection of ctDNA from lung metastasis. **B** Detection of ctDNA from liver metastasis

In this retrospective study, the gene mutation landscape in metastatic breast cancer was compared across two different populations. The results of the mutational analysis and prevalence varied for particular alterations between the two ethnic groups. Currently, cfDNA-derived gene mutational assays have wide application in clinical practice for breast cancer (Fig. 4 A and B). In prior work, the cfDNA-derived mutations were detected using next-generation sequencing (NGS) platforms in almost all of the studies. However, the means of extracting, processing, and analyzing cfDNA samples were different, which can lead to a limited ability to compare differences across populations.

This retrospective, multicenter study is the first proof-of-concept study to report on the use of a single harmonized ctDNA assay in different populations of initially HR+/HER2− MBC from two institutions in the US and CN. The populations analyzed included first-line MBC and primary stage IV BC patients who had not been exposed to treatment in the advanced setting, but who had received similar adjuvant endocrine therapy regimens following standard-of-care guidelines. The study demonstrates that cfDNA analysis can provide a reliable *real-world* assessment of the molecular landscape of metastatic breast cancer and identify differences in genomic abnormalities between patients in the US and CN.

In this study we evaluated a number of cancer variants that are associated with resistance to endocrine therapy alone or in combination with or without PI3K/AKT/mTOR or CDK4/6 inhibitors, such as PTEN gene copy number loss and *ESR1* activating mutations and copy number gain. Previous cfDNA-based studies have reported *PIK3CA* mutations in 18–40% BC patients [26], with *PIK3CA* mutations detected in 43.3% [12] and 25% [27] MBC patients in different studies. In China, the prevalence of *PIK3CA* was 51.3% (40/78) in MBC patients [28] and 46.5% (236/507) in primary BC patients [29]. In this study, the *PIK3CA* mutation prevalence between US and CN patients showed no significant difference in total cohort (*P* = 0.074) or in recurrent subgroup (*P* = 0.179) and in primary stage IV BC subgroup (*P* = 0.069). However, a differential prevalence with statistical significance for *AKT1* (*P* = 0.008) was found between US and CN cohort, respectively. The similar result also observed in first-line MBC subgroup (*P* = 0.001).

In prior work, a mutational frequency of 0.4% was observed for *ATM* alterations in 7,675 *BRCA1* and *BRCA2*-negative breast cancer patients in a Chinese population [30, 31] and a mutational frequency range of 0.45 to 1.0% was found in the US and Europe [32, 33]. However, no available data regarding comprehensive ctDNA-based comparisons between US and CN patients have been reported. Here, we found that *ATM* loss was more frequently observed in CN patients with initially HR+/HER2− MBC as compared to those in the US (16.9% vs. 0.0%, *P* = 0.017). Meanwhile, the high prevalence of *ATM* loss in CN patients was also confirmed in recurrent MBC subgroup (*P* = 0.028). Similar to previous studies [21, 34], our data showed that the mutational prevalence of *CDH1* detected in initially HR+/HER2− MBC was higher in US than CN patients (18.5% vs. 1.5%). Our data also showed the prevalence of *FGFR1* copy number gain was higher in CN patients compared to US patients (*P* = 0.048). This result was in line with previous reports found in HR+/HER2− cohorts of US (1.0 ~ 8.0%) and CN (13.0%) patients [12, 21, 34].

*PTEN* exerts its function in multiple ways including repressing tumor cell growth and cell survival. Nuclear *PTEN* exhibits phosphatase-independent tumor suppressive function such as regulation of chromosome stability, DNA repair, and apoptosis [35, 36]. *PTEN* loss has been identified in cfDNA of 25% MBC patients [27]. Here, we found that the difference in *PTEN* mutation frequency between the US and CN did not reach statistical significance (*P* = 0.15). However, *PTEN* deletion was associated with shorter PFS of HR+ MBC patients in CN (*P* = 0.03), but not in the US (*P* = 0.65).

*ESR1* mutations are associated with poor prognosis in ER+ MBC patients [37]. Chandarlapaty et al. reported that cfDNA *ESR1* mutations were associated with shorter OS of MBC patients from the BOLERO-2 study [10]. A recent meta-analysis by Zhang et al. demonstrated that plasma *ESR1* mutation carriers had significantly worse PFS compared to wild-type ESR1 [38]. In the present study, the ESR1 mutation prevalence between the US and CN was not significantly different. Patients with cfDNA-based *ESR1* copy number gain or mutation had shorter PFS compared to the other patients in the first-line treatment after relapse in the CN patient cohort (*P* = 0.023). However, this finding was not observed in US patients (*P* = 0.62), which may be due to the treatment of most ESR1 mutation carriers with fulvestrant (3/4, 75.0%). The differential predictive value of *PTEN* deletion found in the US vs. CN cohort raised the possibility that in patients receiving a CDK4/6 inhibitor-based regimen (US cohort), the *PTEN* variation may have less effect on the PFS. This finding needs to be evaluated in a larger cohort. Multivariate analysis showed that patients subjected to CDK4/6 inhibitor had longer PFS than patients receiving endocrine therapy only (*P* = 0.02). This result underscores the urgent need for CDK4/6 inhibitor treatment for Chinese patients, since all the patients without CDK4/6 inhibitors were found in the CN cohort. Moreover, there was a trend that patients with liver or lung metastasis tended to have shorter PFS (*P* = 0.18) in the CN cohort, which is not found in the US cohort (*P* = 0.80). This may be partly due to the massive application of CDK4/6 inhibitor in US patients, and this result also needs to be further demonstrated in larger cohort. In addition, the lung metastatic rate was higher in the CN group as compared to the US group (*P* = 0.021). A previous study demonstrated that HR-positive patients with non-visceral metastases had a better prognosis than those with visceral metastases [39]. Thus, we believe the higher lung metastatic rate of the CN group may contribute to the shortened PFS. This study has several limitations. Firstly, this is a retrospective study, with the limitations associated with this type of study. Secondly, the number of patients in each study cohort is relatively small and a larger study is required to more conclusively confirm our finding. Thirdly, it should point out that the study cohort is mixed with first-line MBC and primary stage IV BC. The application of adjuvant/neoadjuvant therapy might also affect the ctDNA landscape of first-line MBC patients. Finally, although we have considered the first-line regimen in multivariate analysis regarding PFS, the significant differences of first-line regimens and re-staging standard between US and CN cohort still cannot be neglected. A larger cohort specifically designed for first-line MBC and primary stage IV BC should be carried out separately to retest our finding in the future works.

## Conclusion

Collectively, our data revealed the differential prevalence of *FGFR1*, *AKT1*, *CDH1*, and *ATM* alterations in initially HR+/HER2− MBC in the US vs. CN patients. In addition, *PTEN* deletion appears to have less effect on PFS in patients receiving CDK4/6 inhibitor-based regimens, a finding that needs to be validated in a larger cohort.

## Supplementary Information

Below is the link to the electronic supplementary material.

Supplementary file1 (PDF 1053 kb)

Supplementary file2: Multivariate analysis containing mutations and patient initial status in the US and CN cohort (DOCX 14 kb)

Supplementary file3: The Gene list of 152-gene PredicineCareTM liquid biopsy assay (DOCX 15 kb)

## Data Availability

All data in the main text are publicly available after its publication. The supporting data of this study are available on request from the corresponding author (Huiping Li).

## References

[1] Bertucci F, Ng CKY, Patsouris A, Droin N, Piscuoglio S, Carbuccia N, Soria JC, Dien AT, Adnani Y, Kamal M, Garnier S, Meurice G, Jimenez M, Dogan S, Verret B, Chaffanet M, Bachelot T, Campone M, Lefeuvre C, Bonnefoi H, Dalenc F, Jacquet A, De Filippo MR, Babbar N, Birnbaum D, Filleron T, Le Tourneau C, Andre F (2019). Genomic characterization of metastatic breast cancers. Nature.

[2] Andre F, Ciruelos E, Rubovszky G, Campone M, Loibl S, Rugo HS, Iwata H, Conte P, Mayer IA, Kaufman B, Yamashita T, Lu YS, Inoue K, Takahashi M, Papai Z, Longin AS, Mills D, Wilke C, Hirawat S, Juric D, SOLAR-1 Study Group (2019). Alpelisib for PIK3CA-Mutated, hormone receptor-positive advanced breast cancer. N Engl J Med.

[3] Setiawan VW, Monroe KR, Wilkens LR, Kolonel LN, Pike MC, Henderson BE (2009). Breast cancer risk factors defined by estrogen and progesterone receptor status: the multiethnic cohort study. Am J Epidemiol.

[4] Tripathy D, Bardia A, Sellers WR (2017). Ribociclib (LEE011): mechanism of action and clinical impact of this selective cyclin-dependent kinase 4/6 inhibitor in various solid tumors. Clin Cancer Res.

[5] Vasan N, Toska E, Scaltriti M (2019). Overview of the relevance of PI3K pathway in HR-positive breast cancer. Ann Oncol.

[6] Osborne CK, Schiff R (2011). Mechanisms of endocrine resistance in breast cancer. Annu Rev Med.

[7] Keup C, Benyaa K, Hauch S, Sprenger-Haussels M, Tewes M, Mach P, Bittner AK, Kimmig R, Hahn P, Kasimir-Bauer S (2020). Targeted deep sequencing revealed variants in cell-free DNA of hormone receptor-positive metastatic breast cancer patients. Cell Mol Life Sci.

[8] Delmonico L, Alves G, Bines J (2020). Cell free DNA biology and its involvement in breast carcinogenesis. Adv Clin Chem.

[9] Spoerke JM, Gendreau S, Walter K, Qiu J, Wilson TR, Savage H, Aimi J, Derynck MK, Chen M, Chan IT, Amler LC, Hampton GM, Johnston S, Krop I, Schmid P, Lackner MR (2016). Heterogeneity and clinical significance of ESR1 mutations in ER-positive metastatic breast cancer patients receiving fulvestrant. Nat Commun.

[10] Chandarlapaty S, Chen D, He W, Sung P, Samoila A, You D, Bhatt T, Patel P, Voi M, Gnant M, Hortobagyi G, Baselga J, Moynahan ME (2016). Prevalence of ESR1 mutations in cell-free DNA and outcomes in metastatic breast cancer: a secondary analysis of the BOLERO-2 clinical trial. JAMA Oncol.

[11] Moynahan ME, Chen D, He W, Sung P, Samoila A, You D, Bhatt T, Patel P, Ringeisen F, Hortobagyi GN, Baselga J, Chandarlapaty S (2017). Correlation between PIK3CA mutations in cell-free DNA and everolimus efficacy in HR(+), HER2(-) advanced breast cancer: results from BOLERO-2. Br J Cancer.

[12] O'Leary B, Cutts RJ, Liu Y, Hrebien S, Huang X, Fenwick K, Andre F, Loibl S, Loi S, Garcia-Murillas I, Cristofanilli M, Huang Bartlett C, Turner NC (2018). The genetic landscape and clonal evolution of breast cancer resistance to palbociclib plus fulvestrant in the PALOMA-3 Trial. Cancer Discov.

[13] Li H, Xu Y, Zhao F, Song G, Rugo HS, Zhang Y, Yang L, Liu X, Shao B, Yang L, Liu Y, Ran R, Zhang R, Guan Y, Chang L, Yi X (2018). Plasma PIK3CA ctDNA specific mutation detected by next generation sequencing is associated with clinical outcome in advanced breast cancer. Am J Cancer Res.

[14] Song QK, Li J, Huang R, Fan JH, Zheng RS, Zhang BN, Zhang B, Tang ZH, Xie XM, Yang HJ, He JJ, Li H, Li JY, Qiao YL, Chen WQ (2014). Age of diagnosis of breast cancer in china: almost 10 years earlier than in the United States and the European Union. Asian Pac J Cancer Prev.

[15] Chen C, Zhou Q, Wu R, Li B, Chen Q, Zhang X, Shi C (2019). A comprehensive survey of genomic alterations in gastric cancer reveals recurrent neoantigens as potential therapeutic targets. Biomed Res Int.

[16] Chen L, Yang L, Yao L, Kuang XY, Zuo WJ, Li S, Qiao F, Liu YR, Cao ZG, Zhou SL, Zhou XY, Yang WT, Shi JX, Huang W, Hu X, Shao ZM (2018). Characterization of PIK3CA and PIK3R1 somatic mutations in Chinese breast cancer patients. Nat Commun.

[17] Li S, Wang X, Li Y, Lai H, Liu Y, Jin L (2019). Non-invasive analysis of tumor mutation profiles and druggable mutations by sequencing of cell free DNA of Chinese metastatic breast cancer patients. Thorac Cancer.

[18] Tao J, Zhang P, Liu G, Yan H, Bu X, Ma Z, Wang N, Wang G, Jia W (2009). Cytotoxicity of Chinese motherwort (YiMuCao) aqueous ethanol extract is non-apoptotic and estrogen receptor independent on human breast cancer cells. J Ethnopharmacol.

[19] Jiang YZ, Ma D, Suo C, Shi J, Xue M, Hu X, Xiao Y, Yu KD, Liu YR, Yu Y, Zheng Y, Li X, Zhang C, Hu P, Zhang J, Hua Q, Zhang J, Hou W, Ren L, Bao D, Li B, Yang J, Yao L, Zuo WJ, Zhao S, Gong Y, Ren YX, Zhao YX, Yang YS, Niu Z, Cao ZG, Stover DG, Verschraegen C, Kaklamani V, Daemen A, Benson JR, Takabe K, Bai F, Li DQ, Wang P, Shi L, Huang W, Shao ZM (2019). Genomic and transcriptomic landscape of triple-negative breast cancers: subtypes and treatment strategies. Cancer Cell.

[20] Huang X, Dugo M, Callari M, Sandri M, De Cecco L, Valeri B, Carcangiu ML, Xue J, Bi R, Veneroni S, Daidone MG, Menard S, Tagliabue E, Shao Z, Wu J, Orlandi R (2015). Molecular portrait of breast cancer in China reveals comprehensive transcriptomic likeness to Caucasian breast cancer and low prevalence of luminal A subtype. Cancer Med.

[21] Zhang G, Wang Y, Chen B, Guo L, Cao L, Ren C, Wen L, Li K, Jia M, Li C, Mok H, Chen X, Wei G, Lin J, Zhang Z, Hou T, Han-Zhang H, Liu C, Liu H, Liu J, Balch CM, Meric-Bernstam F, Liao N (2019). Characterization of frequently mutated cancer genes in Chinese breast tumors: a comparison of Chinese and TCGA cohorts. Ann Transl Med.

[22] Allison KH, Hammond MEH, Dowsett M, McKernin SE, Carey LA, Fitzgibbons PL, Hayes DF, Lakhani SR, Chavez-MacGregor M, Perlmutter J, Perou CM, Regan MM, Rimm DL, Symmans WF, Torlakovic EE, Varella L, Viale G, Weisberg TF, McShane LM, Wolff AC (2020). Estrogen and progesterone receptor testing in breast cancer: ASCO/CAP guideline update. J Clin Oncol.

[23] Davis AA, Zhang Q, Gerratana L, Shah AN, Zhan Y, Qiang W, Finkelman BS, Flaum L, Behdad A, Gradishar WJ, Platanias LC, Cristofanilli M (2019). Association of a novel circulating tumor DNA next-generating sequencing platform with circulating tumor cells (CTCs) and CTC clusters in metastatic breast cancer. Breast Cancer Res.

[24] Gerratana L, Zhang Q, Shah AN, Davis AA, Zhang Y, Wehbe F, Qiang W, Flaum L, Finkelman BS, Gradishar WJ, Platanias LC, Behdad A, Cristofanilli M (2020). Performance of a novel Next Generation Sequencing circulating tumor DNA (ctDNA) platform for the evaluation of samples from patients with metastatic breast cancer (MBC). Crit Rev Oncol Hematol.

[25] Fernandez-Garcia D, Hills A, Page K, Hastings RK, Toghill B, Goddard KS, Ion C, Ogle O, Boydell AR, Gleason K, Rutherford M, Lim A, Guttery DS, Coombes RC, Shaw JA (2019). Plasma cell-free DNA (cfDNA) as a predictive and prognostic marker in patients with metastatic breast cancer. Breast Cancer Res.

[26] Barbareschi M, Buttitta F, Felicioni L, Cotrupi S, Barassi F, Del Grammastro M, Ferro A, Dalla Palma P, Galligioni E, Marchetti A (2007). Different prognostic roles of mutations in the helical and kinase domains of the PIK3CA gene in breast carcinomas. Clin Cancer Res.

[27] Razavi P, Dickler MN, Shah PD, Toy W, Brown DN, Won HH, Li BT, Shen R, Vasan N, Modi S, Jhaveri K, Caravella BA, Patil S, Selenica P, Zamora S, Cowan AM, Comen E, Singh A, Covey A, Berger MF, Hudis CA, Norton L, Nagy RJ, Odegaard JI, Lanman RB, Solit DB, Robson ME, Lacouture ME, Brogi E, Reis-Filho JS, Moynahan ME, Scaltriti M, Chandarlapaty S (2020). Alterations in PTEN and ESR1 promote clinical resistance to alpelisib plus aromatase inhibitors. Nat Cancer.

[28] Chen Z, Zheng Y, Cao W, Zhang Y, Zhao Z, Wang G, Zhao J, Cai S, Shao X, Huang J, Ye W, Huang Y, Li W, Huang X, Wu H, Wang X, Yin Y (2019). Everolimus-containing therapy vs conventional therapy in the treatment of refractory breast cancer patients with PI3K/AKT/mTOR mutations: a retrospective study. Cancer Med.

[29] Deng L, Zhu X, Sun Y, Wang J, Zhong X, Li J, Hu M, Zheng H (2019). Prevalence and prognostic role of PIK3CA/AKT1 mutations in chinese breast cancer patients. Cancer Res Treat.

[30] Yang Z, Ouyang T, Li J, Wang T, Fan Z, Fan T, Lin B, Zhang J, Xie Y (2019). Prevalence and characterization of ATM germline mutations in Chinese BRCA1/2-negative breast cancer patients. Breast Cancer Res Treat.

[31] Liu X, Li H, Shao B, Wu J, Kong W, Song G, Jiang H, Wang J, Wan F (2017). Identification of recurrent BRCA1 mutation and its clinical relevance in Chinese Triple-negative breast cancer cohort. Cancer Med.

[32] Buys SS, Sandbach JF, Gammon A, Patel G, Kidd J, Brown KL, Sharma L, Saam J, Lancaster J, Daly MB (2017). A study of over 35,000 women with breast cancer tested with a 25-gene panel of hereditary cancer genes. Cancer.

[33] Couch FJ, Shimelis H, Hu C, Hart SN, Polley EC, Na J, Hallberg E, Moore R, Thomas A, Lilyquist J, Feng B, McFarland R, Pesaran T, Huether R, LaDuca H, Chao EC, Goldgar DE, Dolinsky JS (2017). Associations between cancer predisposition testing panel genes and breast cancer. JAMA Oncol.

[34] Chung JH, Pavlick D, Hartmaier R, Schrock AB, Young L, Forcier B, Ye P, Levin MK, Goldberg M, Burris H, Gay LM, Hoffman AD, Stephens PJ, Frampton GM, Lipson DM, Nguyen DM, Ganesan S, Park BH, Vahdat LT, Leyland-Jones B, Mughal TI, Pusztai L, O'Shaughnessy J, Miller VA, Ross JS, Ali SM (2017). Hybrid capture-based genomic profiling of circulating tumor DNA from patients with estrogen receptor-positive metastatic breast cancer. Ann Oncol.

[35] Barroso-Sousa R, Keenan TE, Pernas S, Exman P, Jain E, Garrido-Castro AC, Hughes M, Bychkovsky B, Umeton R, Files JL, Lindeman NI, MacConaill LE, Hodi FS, Krop IE, Dillon D, Winer EP, Wagle N, Lin NU, Mittendorf EA, Van Allen EM, Tolaney SM (2020). Tumor Mutational burden and PTEN alterations as molecular correlates of response to PD-1/L1 blockade in metastatic triple-negative breast cancer. Clin Cancer Res.

[36] Dillon LM, Miller TW (2014). Therapeutic targeting of cancers with loss of PTEN function. Curr Drug Targets.

[37] Liao H, Huang W, Pei W, Li H (2020). Detection of ESR1 Mutations Based on Liquid Biopsy in Estrogen Receptor-Positive Metastatic Breast Cancer: Clinical Impacts and Prospects. Front Oncol.

[38] Zhang K, Hong R, Xu F, Xia W, Kaping L, Qin G, Zheng Q, Lu Q, Shi YX, Yuan ZY, Wang S (2018). Clinical value of circulating ESR1 mutations for patients with metastatic breast cancer: a meta-analysis. Cancer Manag Res.

[39] Ozawa H, Sata A, Fukui R, Bun A, Higuchi T, Fujimoto Y, Miyagawa Y, Imamura M, Miyoshi Y (2019). A single-centre, retrospective, observational analysis of fulvestrant for recurrent/metastatic breast cancer according to metastatic site. Anticancer Res.

