# Supplementary material for: Cell-free DNA comparative analysis of the genomic landscape of first-line hormone receptor-positive metastatic breast cancer from the US and China
Source: Breast Cancer Res Treat. 2021 Sep 1;190(2):213–26. doi: 10.1007/s10549-021-06370-w (PMC8558197; doi:10.1007/s10549-021-06370-w)
Supplement: Supplementary file 1 — Supplementary file1 (PDF 1053 kb) [file 10549_2021_6370_MOESM1_ESM.pdf]

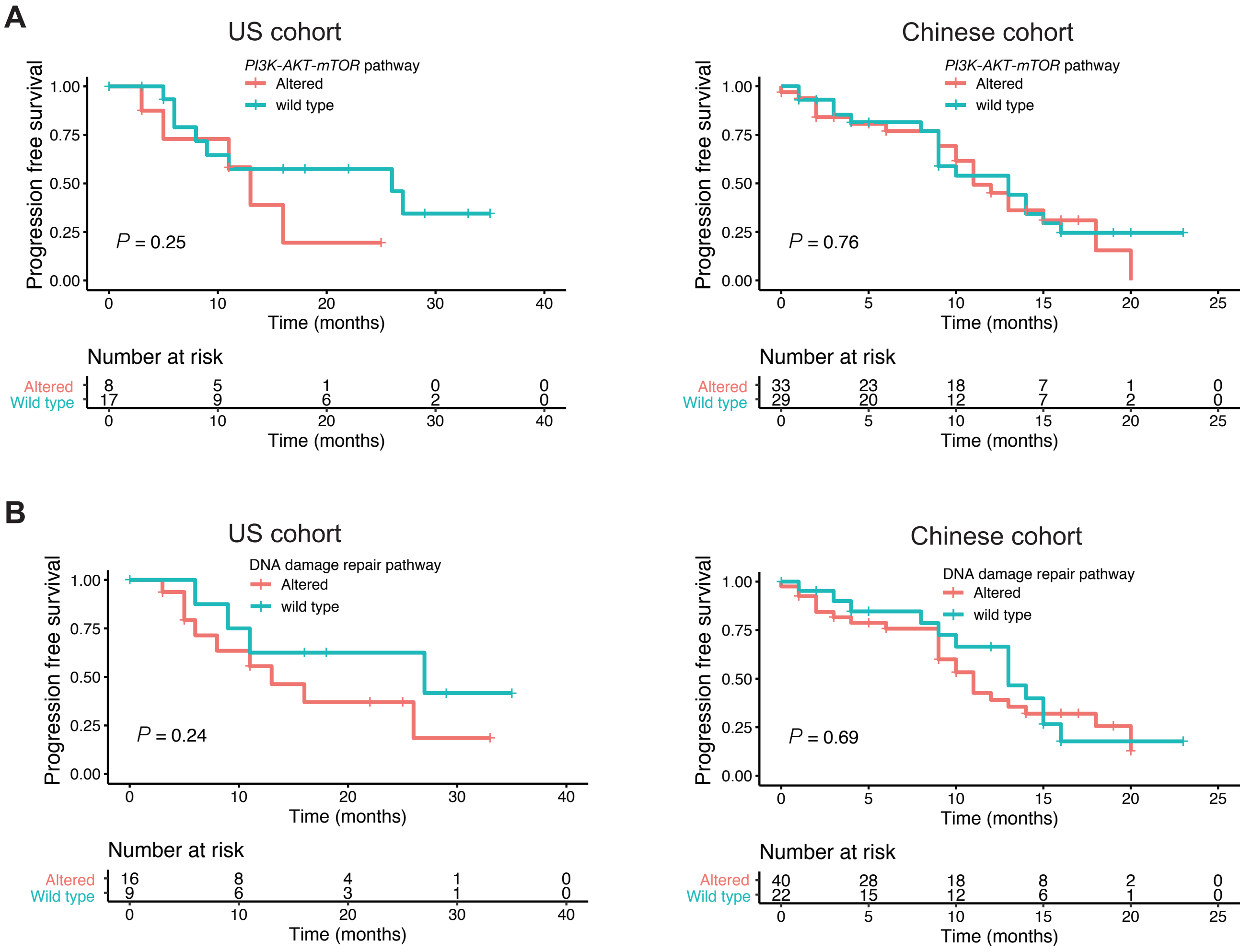

Supplementary figure 1. Survival analysis of Chinese and US patients. **A,** Progression free survival (PFS) of patients with or without *PI3K-AKT-mTOR* pathway alteration. **B,** PFS of patients with or without DNA damage repair pathway alteration. *P* value was calculated using log-rank test.

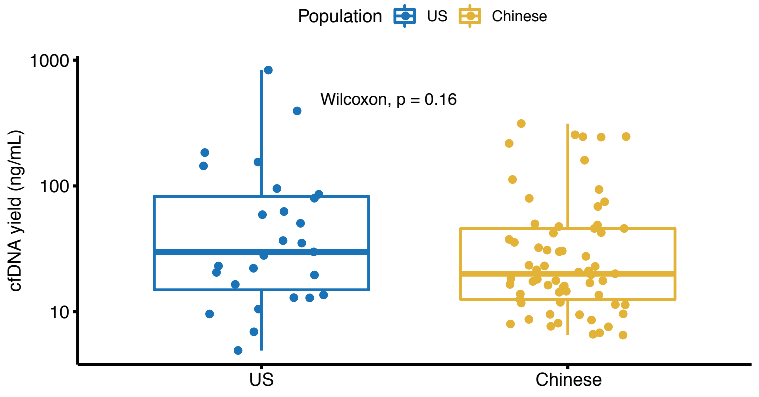

Supplementary figure 2. cfDNA yield between US and Chinese patients. *P* value was calculated using Wilcoxon test.
