# Supplementary material for: Cell-free DNA comparative analysis of the genomic landscape of first-line hormone receptor-positive metastatic breast cancer from the US and China
Source: Breast Cancer Res Treat. 2021 Sep 1;190(2):213–26. doi: 10.1007/s10549-021-06370-w (PMC8558197; doi:10.1007/s10549-021-06370-w)
Supplement: Supplementary file 2 — Supplementary file2: Multivariate analysis containing mutations and patient initial status in the US and CN cohort (DOCX 14 kb) [file 10549_2021_6370_MOESM2_ESM.docx]

| **Multivariate analysis in CN cohort** | |  |  | **Multivariate analysis in CN cohort** | |  |
| --- | --- | --- | --- | --- | --- | --- |
| **Variables** | **HR (95% CI)** | ***p-value*** |  | **Variables** | **HR (95% CI)** | ***p-value*** |
| **Patient initial status** Recurred vs. Denovo | 2.08 (0.81-5.39) | 0.13 |  | **Patient initial status** Recurred vs. Denovo | 2.22 (0.86-5.73) | 0.1 |
| **ESR1** Altered vs Wild type | 2.40 (1.04-5.54) | 0.04 |  | **PTEN** Loss vs Wild type | 3.50 (1.04-11.82) | 0.04 |
|  |  |  |  |  |  |  |
| **Multivariate analysis in US cohort** | |  |  | **Multivariate analysis in US cohort** | |  |
| **Variables** | **HR (95% CI)** | ***p-value*** |  | **Variables** | **HR (95% CI)** | ***p-value*** |
| **Patient initial status** Recurred vs. Denovo | 6.10 (0.71-52.68) | 0.1 |  | **Patient initial status** Recurred vs. Denovo | 6.13 (0.71-52.75) | 0.1 |
| **ESR1** Altered vs Wild type | 1.20 (0.25-5.81) | 0.82 |  | **PTEN** Loss vs Wild type | 1.28 (0.16-10.27) | 0.82 |
|  |  |  |  |  |  |  |
| HR, hazards ratio; CI, confidence interval | |  |  |  |  |  |
