# Supplementary material for: Cell-free DNA comparative analysis of the genomic landscape of first-line hormone receptor-positive metastatic breast cancer from the US and China
Source: Breast Cancer Res Treat. 2021 Sep 1;190(2):213–26. doi: 10.1007/s10549-021-06370-w (PMC8558197; doi:10.1007/s10549-021-06370-w)
Supplement: Supplementary file 3 — Supplementary file3: The Gene list of 152-gene PredicineCareTM liquid biopsy assay (DOCX 15 kb) [file 10549_2021_6370_MOESM3_ESM.docx]

| **Gene list of PredicineCARE panel** | | | | | | | | | |
| --- | --- | --- | --- | --- | --- | --- | --- | --- | --- |
| **Entire coding sequence and CNVs** | | | | | | | | | |
| ABRAXAS1 | AKT1 | AKT2 | AKT3 | ALK | APC | AR | ARAF | ARID1A | ATM |
| ATRX | BAP1 | BARD1 | BCL2 | BRAF | BRCA1 | BRCA2 | BRIP1 | BTK | CCND1 |
| CCND2 | CCND3 | CCNE1 | CCNE2 | CD274 (PD-L1) | CDH1 | CDK12 | CDK2 | CDK4 | CDK6 |
| CDKN2A | CHEK1 | CHEK2 | CTNNB1 | CXCR4 | CYP2C19 | CYP2D6 | CYP3A4 | DAXX | DDR2 |
| DPYD | E2F1 | EGFR | EPCAM | ERBB2 (HER2) | ERBB3 | ERCC1 | ESR1 | EZH2 | FANCA |
| FANCC | FANCF | FANCG | FANCL | FAT1 | FBXW7 | FEN1 | FGFR1 | FGFR2 | FGFR3 |
| FGFR4 | FLT3 | FOXA1 | FOXL2 | FZR1 | GEN1 | GNA11 | GNAQ | GNAS | GSTP1 |
| HNF1A | HOXB13 | HRAS | IDH1 | IDH2 | JAK2 | JAK3 | KDM6A | KIT | KMT2C |
| KMT2D (MLL2) | KRAS | MAP2K1 (MEK1) | MAP2K2 (MEK2) | MAPK1 | MAPK3 | MDM2 | MET | MLH1 | MPL |
| MRE11 | MSH2 | MSH6 | MTHFR | MTOR | MYC | MYCN | MYD88 | NBN | NF1 |
| NFE2L2 | NOTCH1 | NPM1 | NRAS | NTRK1 | NTRK2 | NTRK3 | PALB2 | PDCD1LG2 (PD-L2) | PDGFRA |
| PIK3CA | PIK3CB | PIK3R1 | PLCG2 | PMS2 | POLD1 | POLE | PPP2R1A | PRKACA | PRKD1 |
| PTEN | PTPN11 | RAD50 | RAD51 | RAD51B | RAD51C | RAD51D | RAD52 | RAF1 | RB1 |
| RET | RHEB | RHOA | RIT1 | RNF43 | ROS1 | SDHB | SMAD4 | SMO | SPOP |
| STAG2 | STK11 | TERT promoter | TMPRSS2 | TP53 | TSC1 | TSC2 | UGT1A1 | VHL | XPC |
| XRCC1 |  |  |  |  |  |  |  |  |  |
| **Fusions** | | | | | | | | | |
| ALK | BRAF | CD274 | CD74 | EGFR | FGFR1 | FGFR2 | FGFR3 | NTRK1 | NTRK2 |
| PDGFRA | PRKACA | RET | ROS1 | TMPRSS2 |  |  |  |  |  |
